# Epidemiology of COVID-19 in the State of Sergipe/Brazil and Its Relationship with Social Indicators

**DOI:** 10.3390/epidemiologia2030020

**Published:** 2021-07-14

**Authors:** Larissa M. Fonseca, Derijuli S. de Sousa, Juliana C. Cardoso, Patricia Severino, Amanda Cano, Eliana B. Souto, Sônia O. Lima, Cristiane C. C. de Oliveira, Francisco P. Reis

**Affiliations:** 1 Post-Graduation Program in Health and Environment, University of Tiradentes, Aracaju 49010-390, Sergipe, Brazil; larissa.marrocos@gmail.com (L.M.F.); deriartur@gmail.com (D.S.d.S.); juliana_cordeiro@itp.org.br (J.C.C.); sonialima.cirurgia@gmail.com (S.O.L.); criscunhaoliva@yahoo.com.br (C.C.C.d.O.); 2 Institute of Technology and Research (ITP), University of Tiradentes, Aracaju 49010-390, Sergipe, Brazil; pattypharma@gmail.com; 3 Post-Graduation Program in Biotechnology, University of Tiradentes, Aracaju 49010-390, Sergipe, Brazil; 4 Department of Pharmacy, Pharmaceutical Technology and Physical Chemistry, Faculty of Pharmacy and Food Sciences, University of Barcelona, 08028 Barcelona, Spain; acanofernandez@ub.edu; 5 Networking Research Centre of Neurodegenerative Disease (CIBERNED), Instituto de Salud Juan Carlos III, 28031 Madrid, Spain; 6 Department of Pharmaceutical Technology, Faculty of Pharmacy, University of Coimbra, 3000-548 Coimbra, Portugal; 7 CEB—Centre of Biological Engineering, University of Minho, 4710-057 Braga, Portugal

**Keywords:** Coronavirus infections, COVID-19, development indicators, mortality, Sergipe/Brazil, epidemiology

## Abstract

A pandemic is capable of generating a great impact, not only from the point of view of health, but also socioeconomically. In March 2020, the World Health Organization (WHO) declared that a new pandemic situation had arisen, due to the SARS-CoV-2 virus, whose probable origin was zoonotic. The largest number of cases of this disease is concentrated in the United States of America (USA), India, and Brazil. The mortality rate is estimated at 3.4%, but regional differences may exist, and places with a high demographic density have become true epicentres and may be related to higher rates of transmission. In addition to the above, lower human development indexes (HDI) can be related to worse outcomes, especially in the North and Northeast regions of Brazil since they are the least developed places. The Northeast region is the second-most-affected place in the number of COVID-19 cases in Brazil. An analytical observational study of an ecological type was carried out from April to October 2020 to assess the epidemiological situation of COVID-19 in the state of Sergipe and specifically to analyse the incidence of cases and deaths resulting from COVID-19 in the different health regions of the state of Sergipe, in relation to the values of the HDI and demographic density. During the study period, 84,325 cases of COVID-19 were identified, in which 2205 resulted in death. In most of the regions studied, there was a positive association between the number of cases and deaths and the greater the demographic density, but there was no increase in the risk of becoming ill, nor of dying the lower the HDI. Large and crowded cities are places of greatest vulnerability to illness, due to their greater capacity of transmitting the virus; however, further studies are needed to identify other factors that are decisive in the outcomes of this new disease.

## 1. Introduction

In March 2020, a new pandemic situation was declared, caused by the new coronavirus [1]. CoV-type viruses belong to a family of RNA, encapsulated, with a structure similar to a crown, and are subdivided into 4 groups (Alphacoronavirus, Betacoronavirus, Gammacoronavirus, and Deltacoronavirus). This family has been responsible for two human infections in the past: SARS-CoV in 2002 in China, and the MERS–CoV infection (Middle East Respiratory Syndrome) in Saudi Arabia in 2012. Both were related to animal reservoirs that emerged and became able to infect humans with acute respiratory disease. Due to the similarity with SARS-CoV, the new coronavirus was called SARS-CoV-2 [1].

As of 17 January, 2021, 95,179,173 positive cases and 2,044,445 deaths have been identified worldwide. The countries with the highest number of cases are the United States (USA), India, and Brazil, respectively, and in number of deaths, USA, Brazil, and India. The worldwide mortality rate is estimated at 3.4%, but it has regional differences, being higher in Mexico (9.9%), Iran (5.7%) and Italy (5.0%), and lower in countries such as Singapore (tending to 0%), Sri Lanka (0.2%) and Qatar (0.2%), countries with different regional characteristics and different human development indices (HDI) [2,3].

In Brazil, up to that date, 8,488,099 cases of coronavirus were registered and, of these, 210,299 died due to this pathology. In the state of Sergipe, according to the State Secretariat, 128,395 cases of infection by SARS-CoV-2 and 2662 deaths were identified, with the first case report of this infection in March of 2020 [4,5].

The Northeast is the Brazilian region that has the second-highest rate of number of confirmed cases of COVID-19, mainly in the states of Ceará, Bahia, Pernambuco, and Rio Grande do Norte. It is also noteworthy that the lethality in some states in the Northeast (such as Pernambuco, Paraíba, and Piauí) is higher than the average reported by the WHO (3.4%) and higher than the Brazilian average—5.4%, being respectively, 4%, 5% and 22.2% [6].

The HDI is a measure that represents economic issues (through per capita income), and also aspects of human life through the analysis of local health and educations, and is the main measure of human development worldwide through which public policies are developed. Studies show that there is a strong relationship between the HDI and the quality of local health. It has a range from 0 to 1, where the first represents no development and the second represents total human development. Sites with an HDI below 0.499 have a development that is considered low, between 0.500 and 0.799 is considered to be medium development and, finally, places with an HDI above 0.800 are considered to have high development. Low HDI locations may be associated with higher mortality and vulnerability to acquiring COVID-19 [7,8].

Factors such as social inequality, poor housing conditions, low income, demographic structure, lack of family structure, and previous living conditions, place such places as the most vulnerable to the development of more severe forms of the disease [9].

The poorest countries are also affected by COVID-19, since they already live with chronic diseases, as well as difficulties in accessing healthcare, and with the advent of the pandemic, the already-experienced scenario worsened. In addition to the above, they are populations without economic savings, and many have essentially informal jobs, such as selling products or providing services that have been temporarily suspended [7,10].

The presence of high demographic density and intense urbanization proved to be a determining factor in the rate of transmission of the disease in the countries of Latin America, being more intense in cities where there is a greater agglomeration of people, poorer hygiene conditions, water scarcity, impossibility to promote social distancing, and the impossibility of carrying out hand washing properly, which can contribute to the perpetuation of the disease transmission chain. On the other hand, it is in large cities that the best access to the healthcare system is obtained, which may lead to lower mortality rates [11,12].

The state of Sergipe is the smallest Brazilian state. Its territory is around 21,962.10 km^2^, which corresponds to 0.26% of the national territory and 1.4% of the northeast region. It consists of 75 municipalities, and its capital is the coastal city of Aracaju. Its population was estimated at 2,320,840 inhabitants, which corresponds to 3.8% of the population in the Northeast and 1% of the Brazilian population. It has a demographic density of 94.35 inhabitants per square kilometre and its HDI is 0.665, being, therefore, lower than the national HDI—0.816 [13,14,15].

Knowing that the HDI can evaluate social characteristics that influence human life, it is very useful in analyzing the reality of each location; therefore, there may be worse clinical outcomes resulting from COVID-19 in places with lower social indicators, such as the HDI, due to the high vulnerability of the residents of these places being worse in small cities [7,16].

The state of Sergipe is divided into seven health regions, which are spaces delimited through cultural, economic, and social identity, thus facilitating the organization, planning, and execution of health services. They are: Nossa Senhora da Glória Region, Propriá Region, Itabaiana Region, Lagarto Region, Nossa Senhora do Socorro Region, Aracaju Region and Estancia Region. The epidemiological profile of COVID-19 in the State of Sergipe was considered to be favourable when compared to the national and world average. The incidence was estimated at 3670/100 thousand inhabitants, and the lethality in this state has remained around 2 to 2.6% being considered, therefore, as low.

Based on the hypothesis that mortality, as well as the number of cases, would be higher in the municipalities with the lowest HDI and the highest population densities, the objective of this work was to evaluate the epidemiological situation of COVID-19 in the state of Sergipe and specifically to analyse the incidence of cases and deaths resulting from COVID-19 in the different health regions of the state of Sergipe, in relation to the values of the HDI and demographic density.

## 2. Materials and Methods

We conducted an observational analytical study of an ecological type in which the epidemiological bulletins published online by the State of Sergipe’s Health Secretariat on the situation of COVID-19 in this state were analysed. For the development of this study, there was no need for approval by the ethics committee, since it had secondary data sources (open data) already available for online access. It was also not necessary to use the Free and Informed Consent Term (ICF) because there was no direct contact with those surveyed. In this study, the state of Sergipe was divided into clusters for analysis. The health regions of Sergipe were used for these clusters in order to observe the disease behaviour at those localities, since they are divided by similarities between health and economy that may influence COVID-19 rates of incidence and mortality. The population used in this study was obtained by the DataSUS. The incidence (number of new cases divided by the population with risk of falling ill) and number of deaths were compared with the local HDI (online data available through the United Nations Development Program—UNDP). For the analysis, the average HDI of each health region was calculated. To study the impact that high population density has on COVID-19 case numbers and deaths, it was decided to compare these rates with the demographic density. The period studied was from April to October 2020 and has as inclusion criteria all patients who tested positive and died from COVID-19 who live in the state of Sergipe. For the statistical analysis, the negative binomial regression model was used to assess the relationship between the number of deaths and cases in relation to the HDI (10×), the Average Demographic Density of the regions and Lethality once the number of deaths is counted as data with overdispersion [17]. The Beta regression model was also used to assess the relationship between the incidence of deaths and cases against the region’s average HDI (10×) and demographic density, as the incidence of deaths is a percentage [18]. The negative binomial regression gives regression coefficients which could be interpreted as incidence rate ratios, such as Poisson distribution [17], and Beta regression coefficients could be interpreted as odds ratios, such as Logistic regressions [18]. Parameter estimates and their respective intervals were evaluated with 95% confidence per health region. The level of significance adopted was 5% and the software adopted was the R Core Team 2020.

## 3. Results

**Figure 1:**
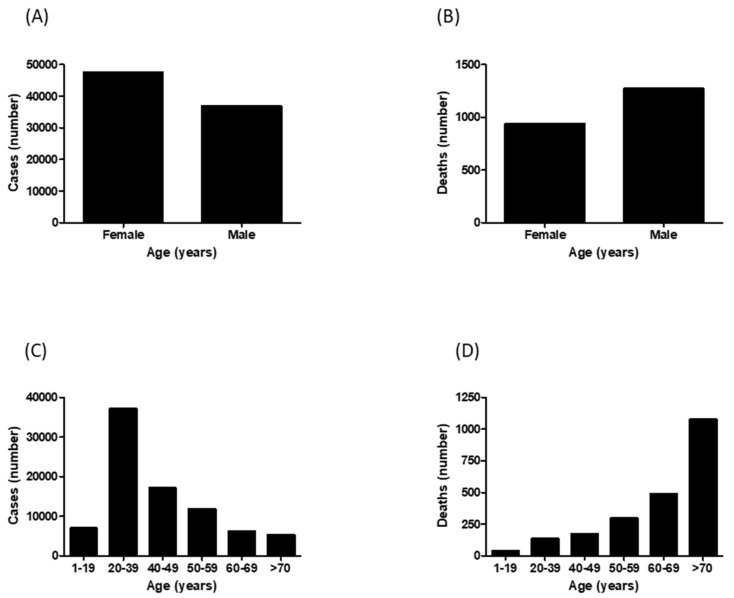
Demographic data of COVID-19 in the state of Sergipe (Brazil) from April to October 2020. (**A**) Distribution of cases between male and female. (**B**) Distribution of deaths between male and female. (**C**) Distribution of cases between different age ranges. (**D**) Distribution of deaths between different age ranges.

During the study period, from April to October 2020, 84,325 cases of COVID-19 in the state of Sergipe were confirmed: 47,478 (56.3%) female and 36,848 (43.7%) male (Figure 1A).

According to the age group, the number of cases, in decreasing order, had the following distribution: 37,040 (43.9%), in the age group of 20 to 39 years, followed by the 40 to 49 years old age group, with 17,041 (20.2%) cases; 50 to 59, 11,779 (14%); less than 19 years, 6957 (8.25%); 60 to 69, 6190 (7.3%); and up to 70 years with 5318 (6.3%) cases (Figure 1C).

During this study period, 2205 deaths were recorded. Unlike what happened in the number of cases, most of these deaths occurred in males, 1270 (57.6%), while in females there were 936 (42.4%) deaths (Figure 1B).

The majority of deaths (48.6%) occurred in the age group of up to 70 years, followed by patients those belonging to the range of 60 to 69 years of age, which corresponded to 22.1%. The least-affected age group was less than 19 years, which corresponded to only 1.8% of deaths (Figure 1D).

**Figure 2:**
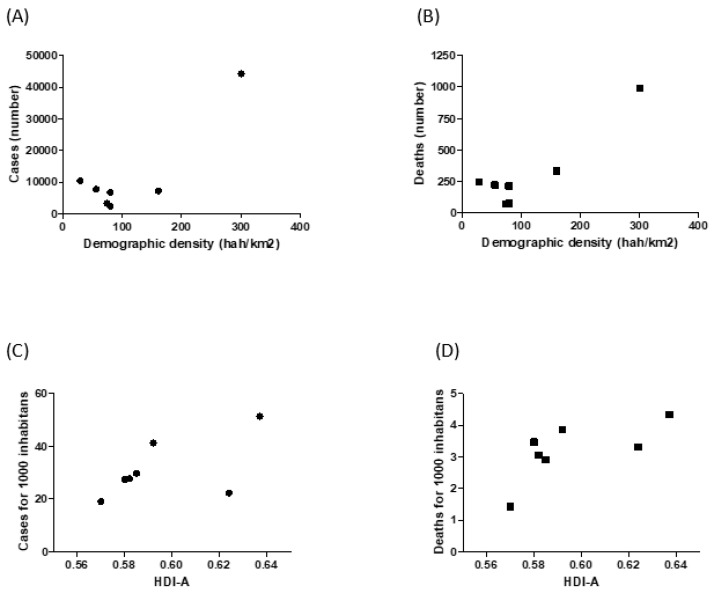
Cumulative data about COVID-19 by health regions of the State of Sergipe, Brazil, from April to October 2020. (**A**) Relationship between demographic density and confirmed COVID-19 cases; (**B**) relationship between demographic density and deaths by COVID-19; (**C**) relationship between COVID-19 case incidence and Human Development Index—average (HDI-A); (**D**) relationship between COVID-19 death incidence and Human Development Index—average (HDI-A).

The number of cases of COVID-19, distributed among the seven health regions of the state of Sergipe, was directly proportional to the value of the demographic density of these regions. The highest concentration of deaths occurred in the Region of Aracaju (Figure 2).

**Figure 3:**
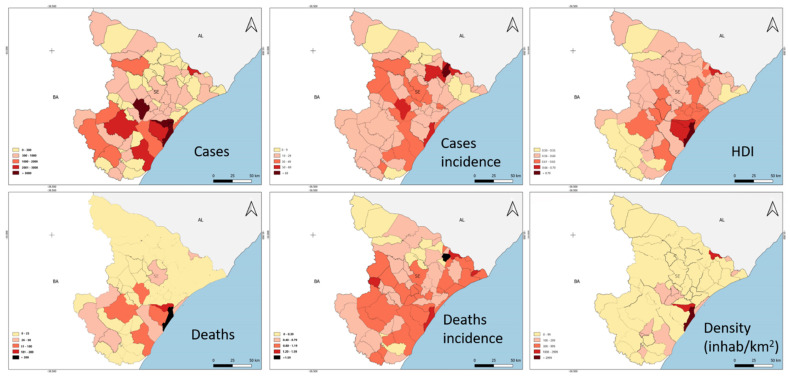
COVID-19 territorial distribution in the State of Sergipe, Brazil, from April to October 2020. Case number, case incidence per 1000 inhabitants, HDI, death number, death incidence per 1000 inhabitants and density (inhabitants/km^2^).

The ratio of the incidence rate (RTI), according to the negative binomial regression applied, showed that in this correlation of the number of cases and demographic density, there was statistical significance in five of the health regions (Table 1). A similar situation occurred in relation to the number of cases and deaths; therefore, there is a positive association in all regions, but with significance in only five regions (Table 2). Figure 3 depicts the COVID-19 territorial distribution in Sergipe (Brazil) from April to October 2020.

Table 3 shows the correlation found between the incidence of cases of COVID-19 and the HDI mean of each of the health regions of the state of Sergipe. The data show, according to the positive association, that there is a greater chance of falling ill in regions with a higher HDI in most of the regions (six), but it is significant in only one region (Estância). The same occurred when the incidence of deaths was correlated with the HDI (Table 4): most of the regions (five) had a higher risk of death in the regions with a higher HDI; however, only two regions (Aracaju and Propriá) had a statistically significant number. 

Table 4 shows that there was a greater chance of dying from COVID-19 in the health regions with the highest HDI, with a value that is also statistically significant for the health region of Aracaju.

## 4. Discussion

The state of Sergipe, even territorially being the smallest state in Brazil, has different realities both in health and socially in its regions. Some municipalities in these regions have HDIs comparable to better-developed countries, and others have really low HDIs. Their HDI means (HDI-Ms) range from 0.570 to 0.637. However, HDI by municipality ranges from 0.529 to 0.77. The region of Aracaju, where the capital of the state is, has the largest HDI-M (0.637), and the smallest is in the Region of Glória. The city of Poço Redondo has the smallest HDI of the state (0.529) [13]. In the same way, the demographic density throughout the state, the capital and a few cities have high density. The lowest density is Gararu (17 inhab/km^2^), located in the Region of Glória and the highest density is on the capital Aracaju (3140 inhab/km^2^).

The findings of the present study regarding mortality by COVID-19 between men and women were similar to those reported in the literature, that men have a higher mortality when compared to women. For such a mortality rate, endocrinological and hormonal factors have been studied. However, other factors, such as early medical care and better life health behaviours (that are more frequent in women), can be considered [19].

As for the correlation between age group and mortality, a direct relationship was found according to an increase in age. [20] The findings of this current study agree with what was carried out in Austria, reporting that mortality was higher in people over 55 to 60 years of age [21], similarly to what was observed in Brazil, reporting that mortality by COVID-19 was higher for older age groups [22].

When the number of cases of COVID 19 was compared with the demographic density of the regions of the state of Sergipe, in the present study, a positive association was obtained, that is, the greater the demographic density, the greater the number of cases, as well as the mortality. This finding is similar to the study carried out in Algeria, which found a strong relationship between demographic density and the number of cases of COVID-19, indicating that the people’s proximity plays a key role in the spread of the virus [23].

In the present study, a positive association was also found between the health regions of the study regarding the incidence of COVID-19 with the HDI. This finding is conflicting with the results of the study carried out in municipalities in Ceará [24]. In this study, it was reported that there was a correlation between a higher incidence of COVID-19 with a low HDI, indicating an important association of COVID-19 with social factors. However, the present study corroborated the results found in another study carried out in the same state—Ceará [25]—in which the higher incidence of COVID-19 that was reported, the higher the HDI. In this situation, the first cases were related to travelling abroad, indicating that high HDIs also contribute to circulation and viral transmission, and it is mandatory to observe the evolution of COVID-19, especially when reaching the social strata of lower HDIs.

The present study is in line with what was expressed by some authors when they stated the need to investigate the form of propagation, as well as to encourage information based on scientific studies, in order to bring knowledge to the population, to develop different lines of action when facing COVID-19, and to strengthen the public health system, especially in poor countries that suffer the greatest damage from the pandemic [12,26,27].

## 5. Conclusions

Mortality due to COVID-19 is strongly present in the health regions of the state of Sergipe, especially in Aracaju. It was possible to verify in this study that demographic density is the main factor for a greater possibility of falling ill due to COVID-19, leading, therefore, to a greater number of infected people and deaths. This was significant in most of regions studied: Aracaju, Nossa Senhora da Glória, Itabaiana, and Nossa Senhora do Socorro. Additionally, the number of deaths by demographic density of the regions was significant in the regions of Aracaju, Itabaiana, Nossa Senhora do Socorro, and Propriá. Statistically, there was a positive relationship between the occurrence of the incidence of cases of COVID-19, as well as the chance of falling ill, in health regions with the highest HDI, confirming that agglomerations and big cities played a fundamental role in the transmission of COVID-19, having more importance than a low HDI in this study. The same occurred when the incidence of deaths was correlated with the HDI. It was found that the chance of dying from COVID-19 was greater in health regions with the highest HDI. In both situations, the numbers were statistically significant for the health region of Aracaju. The SARS-CoV-2 pandemic remains, to date, a public health challenge, not only in Sergipe, Brazil, but around the world. It has reached all social groups, with repercussions not only on health, but also on the economy, education, and people’s behaviour. It is possible that it is the biggest health problem since the influenza pandemic in 1918. Despite the limitations of the present study (ecological type study), it is valid to reiterate that the epidemiological importance opens possibilities for further investigations to guide the disease’s behaviour in the state of Sergipe, and the behaviour of SARS-CoV-2 is observed in this state.

## Figures and Tables

**Table 1:** Ratio of the incidence rate (RTI) between number of COVID-19 cases and demographic density of health regions—State of Sergipe. Period—April to October 2020.

Region	Demographic Density (hab/km^2^)	Number Cases by Region	IRR	IC95%−	IC95%+	*p*-Value
Aracaju	301,150	44,216	1.001	1.001	1.002	<0.001
Itabaiana	28,572	10,442	1.008	1.003	1.014	<0.001
Socorro	55,690	7738	1.002	1.001	1.004	0.001
Lagarto	160,688	7232	1.016	0.992	1.042	0.239
Estância	79,393	6758	1.002	0.991	1.016	0.668
Glória	74,181	3297	1.126	1.051	1.213	<0.001
Propriá	79,765	2445	1.006	1.002	1.011	0.023

Caption: IRR—Incidence Rate Ratio IC95%−+—lower and upper limit with 95% of confidence. Negative Binomial Regression.

**Table 2:** Ratio of the incidence rate (RTI) between number of deaths by COVID-19 with demographic density of the health regions of the State of Sergipe. Period—April to October 2020.

Region	Demographic Density (hab/km^2^)	Number of Deaths by Region	IRR	IC95%−	IC95%+	*p*-Value
Aracaju	301,150	989	1.001	1.001	1.002	<0.001
Lagarto	160,688	221	1.022	1.002	1.044	0.099
Itabaiana	28,572	235	1.008	1.004	1.013	0.004
Socorro	55,690	326	1.003	1.002	1.004	<0.001
Estância	79,393	213	1.000	0.987	1.010	0.952
Propriá	79,765	143	1.007	1.003	1.010	0.002
Glória	74,181	78	1.078	1.010	1.150	0.053

Caption: IRR—Incidence Rate Ratio IC95%−+—lower and upper limit with 95% of confidence. Negative Binomial Regression.

**Table 3:** Relation between incidence of COVID-19 cases and HDI of health regions in Sergipe—April to October 2020.

Region	P	C	I	HDI-M	OR	IC95%−	IC95%+	*p*-Value
Aracaju	860,938	44,216	51.36	0.637	1.241	0.742	2.073	0.410
Itabaiana	252,805	10,442	41.30	0.592	1.588	0.618	4.078	0.337
Propriá	152,916	4538	29.68	0.585	1.965	0.923	4.180	0.079
Lagarto	260,614	7232	27.75	0.582	0.625	0.291	1.339	0.227
Estância	246,282	6758	27.44	0.580	1.989	1.073	3.685	0.029
Socorro	352,006	7843	22.28	0.624	1.405	0.514	3.845	0.508
Glória	173,135	3297	19.04	0.570	1.888	0.343	10.399	0.465

Caption: P—Population. C—Number of cases. I—Incidence of COVID-19 cases per 1000 inhabitants. HDI-M—Human Development Index (Mean). OR—Odds Ratio IC95%−+—lower and upper limit with 95% confidence. Beta Regression.

**Table 4:** Relationship between incidence of deaths by COVID-19 and HDI of health regions in April to October 2020.

Region	P	D	I	HDI-M	OR	IC95%+	IC95%−	*p*-Value
Aracaju	860,938	989	1.14	0.637	1.151	1.007	1.314	0.008
Propriá	152,916	143	0.93	0.585	3.336	1.043	10.671	0.042
Itabaiana	252,805	235	0.92	0.592	1.281	0.591	2.778	0.530
Socorro	352,006	326	0.92	0.624	1.834	0.712	4.725	0.209
Estância	246,282	213	0.86	0.580	1.509	0.725	3.137	0.271
Lagarto	260,614	221	0.84	0.582	0.970	0.695	1.354	0.858
Glória	173,135	78	0.45	0.570	0.101	0.010	1.006	0.051

Caption: P—Population. D—Number of Deaths. I—Incidence of COVID-19 deaths per 1000 inhabitants. HDI-M—Human Development Index (Mean). OR—Odds Ratio. IC95%−+—lower and upper limit with 95% confidence. Beta Regression.

## Data Availability

Not applicable.
